# Nerve Blocks in the Geriatric Patient With Hip Fracture: A Review of the Current Literature and Relevant Neuroanatomy

**DOI:** 10.1177/2151458517734046

**Published:** 2017-10-13

**Authors:** Nirav H. Amin, Jacob A. West, Travis Farmer, Hrayr G. Basmajian

**Affiliations:** 1 Department of Orthopedic Surgery, Loma Linda University Medical Center, Loma Linda, CA, USA; 2 Loma Linda University, Loma Linda, CA, USA; 3 Pomona Valley Hospital Medical Center, Department of Orthopedic Surgery, Pomona, CA, USA

**Keywords:** nerves, pain, fascia iliaca compartment block, nerve block, hip fracture

## Abstract

**Introduction:**

Hip fracture is a common occurrence in the elderly population with high morbidity and mortality due to postoperative pain and opioid use. The goal of this article is to review the current literature on the neuroanatomy of the hip and the use of localized nerve block in controlling hip fracture pain.

**Methods:**

A thorough search of MEDLINE/PubMed, Embase, and the Cochrane Database of Systematic Reviews was conducted using the search terms “hip fracture” and “fascia iliaca block (FICB).” An additional search was conducted utilizing multiple search terms including “hip fracture,” “greater trochanter,” “femur,” “hip,” “anatomy,” “neuroanatomical,” and “anatomic.” Each search result was investigated for cadaveric studies on the innervation of the trochanteric region.

**Results:**

Twenty-five clinical studies examining the use of FICBs in hip fracture patients were identified. These studies show that FICB is safe and effective in controlling perioperative pain. Additionally, FICB has been shown to decrease opioid requirement and opioid-related side effects. Neuroanatomical studies show that the hip capsule is innervated by contributions from the femoral, obturator, sciatic, and superior gluteal nerves. Imaging studies suggest that FICB anesthetizes these branches through localized spread along the fascia iliaca plane. Cadaveric evidence suggests that the greater trochanter region is directly innervated by a single branch from the femoral nerve.

**Discussion:**

The proven efficacy of nerve blocks and their anatomic basis is encouraging to both the anesthesiologist and orthopedic surgeon. Their routine use in the hip fracture setting may improve patient outcomes, given the unacceptably high morbidity and mortality associated with opioid use.

**Conclusions:**

Localized nerve blocks, specifically FICB, have been shown to be safe and effective in managing acute hip fracture pain in geriatric patients, leading to decreased opioid use. Knowledge of the hip neuroanatomy may help guide future development of hip fracture pain blockade.

## Introduction

As the incidence of hip fractures has been increasing, intertrochanteric hip fractures continue to be a common occurrence in the elderly population.^1^ Models currently estimate the incidence of hip fractures by the year 2050 to be as high as 1 037 000 annually, with 35% of these occurring in the intertrochanteric region.^2,3^ These fractures in the elderly population can be debilitating, with 10% of patients never returning to their previous residence, suffering reduced quality of life, and facing mortality rates as high as 1/3 at 1 year, which has largely remained unchanged over the past several decades.^4^ Elderly patients are also at risk of developing postoperative complications secondary to postoperative pain or the methods of pain control. Poor control of postoperative pain can lead to delirium, which is associated with delayed return of functional status, increased mortality, and poor functional outcomes at 6 months postoperatively.^5^

The prevalence of delirium after sustaining hip fracture has been found to be as high as 40% after surgical fixation with evidence of contribution by opioid narcotics.^6^ Lee et al found patients who had dementia or delirium after a hip fracture had nearly a 2-fold greater odds of 1-year mortality.^7^ Despite the known untoward effects of opioid analgesia in a sensitive elderly population, Schepis and McCabe published findings from the National Survey on Drug Use and Health that demonstrated a continued increase in opioid use within the older adult population.^8^ Prescription opioid abuse and misuse within the elderly population lead to increased susceptibility to illness, higher likelihood of altered presence of illness, and impaired recovery.^9^ In an effort to combat the morbidity and mortality associated with delirium, researchers have investigated alternatives to opioids to control pain in elderly hip fracture patients including various nerve blocks^10^ and systemic treatments such as methylprednisolone^11^ and nonsteroidal anti-inflammatory medications (NSAIDs). Freeman and Clarke’s sweeping review of the literature stresses that analgesia in the elderly population should focus on minimizing risk factors for delirium including, pain, constipation, and deliriogenic side effects. They found that fascia iliaca compartment blocks (FICBs) are safe and easy to perform in the elderly population, reduce opioid requirement, and are effective in pain reduction and delirium prevention.^12^ Several other articles have demonstrated the efficacy of the fascia iliac block within the hip fracture cohort to control pain from the fracture site.^13-18^ The success garnered from this research presents an exciting opportunity to both improve pain control and reduce complications in patients with hip fracture. The purpose of this study is to review the current literature on the use of nerve blocks in hip fracture and survey the neural anatomy of the hip to optimize the efficacy of local nerve blocks in the management of hip fracture pain.

## Methods

A search of MEDLINE/PubMed, Embase, and the Cochrane Database of Systemic Reviews was conducted using the search terms “hip fracture” and “fascia iliaca block” to review the current literature on the safety and efficacy of FICB in patients with hip fracture. Each search result was investigated for clinical studies addressing the utilization of fascia iliac blocks in hip fracture patients.

A thorough search was conducted in the current literature utilizing multiple search terms including “hip fracture,” “greater trochanter,” “femur,” “hip,” “anatomy,” “neuroanatomical,” and “anatomic.” Each search result was investigated for cadaveric studies on the innervation of the trochanteric region. The database included MEDLINE/PubMed, Embase, and the Cochrane Database of Systemic Reviews. Three independent reviewers scanned the abstracts, study titles, and key findings to include within the neural mapping. Studies involving the innervation of the hip capsule, periarticular region, and studies not in English were excluded.

## Results

In the literature review, FICB was more commonly related to hip fractures than any other localized nerve block. Twenty-five clinical studies examining the use of FICBs in hip fracture patients were identified. Thirteen of these articles were centered on the preoperative use of FICB in hip fracture patients, 6 studies examined the postoperative use of FICB, and 4 studies compared FICB with femoral nerve block. Two articles were identified comparing the use of FICB with systemic NSAIDs. Finally, 1 study was identified reporting decreased incidence of postoperative delirium in intermediate risk hip fracture patients who received FICB preoperatively.

Only 1 study by Genth et al was identified as a thorough neuroanatomical investigation of the trochanteric region of the hip.^19^ Four other studies were identified pertaining to the neuroanatomical basis of localized nerve blocks in the management of hip fracture. These studies included cadaveric dissections delineating the innervation of the hip, radiographic studies employing computed tomography and magnetic resonance imaging (MRI) to examine the spread of anesthetic agent in the use of various nerve blocks, and a study on the use of ultrasound guidance to administer FICB. One study was also identified comparing the efficacy and anesthetic distribution of FICB and 3-in-1 femoral nerve block.

## Fascia Iliaca Compartment Block Versus Femoral Nerve Block

Anesthesiology literature is ripe with an array of various pain blocks that have proven useful in the treatment of both preoperative and postoperative pain in patients with hip fracture. When evaluating pain control techniques, there seems to be no clear consensus on which block is most effective. In a recent randomized control trial, Newman et al showed a small but significant (*P* = .047) reduction in visual analog scores (VAS) for patients receiving femoral nerve blocks compared to FICB. The authors noted however that femoral nerve blocks were more expensive and required more time to perform.^20^ Temelkovska-Stevanovska et al also found lower pain scores and decreased opioid use in patients receiving continuous femoral nerve block compared to single FICB. The authors suggested that continuous anesthetic through an indwelling catheter is more effective than FICB administered only once.^21^ Several other studies have been published, however, demonstrating no difference in pain scores and morphine requirement between patients treated with FICB and femoral nerve blocks.^22,23^ In examining prolonged nerve block via an indwelling cannula, Yu et al found that continuous FICB produced significantly decreased VAS compared to continuous femoral nerve block.^24^ Additionally, Capdevila et al showed FICB provided a faster and more consistent sensory blockade of the lateral femoral nerve distributions than 3-in-1 blocks.^25^ Although there is conflicting evidence on which nerve block is superior, recent literature has focused on FICB due to its relative ease of administration in the emergency department (ED), low incidence of block failure, and protective effect against delirium in the geriatric population (Table 1).

**Table 1.** Summary of Studies Comparing FICB and 3-in-1 Femoral Nerve Block.

Study	Year	Method	Fracture	Comparison	Primary Outcome	Results
Reavley et al^22^	2015	Prospective randomized	Femoral neck fracture	83 3-in-1 femoral block versus 79 FICB	VAS (100 mm scale)	Mean VAS was 45 mm for 3-in-1 femoral block group versus 44 mm for the FICB group (*P* = .85)
Deniz et al^2^ ^3^	2014	Prospective randomized	Femoral neck	20 FICB versus 20 3-in-1 versus 20 control (GA only)	VAS (0-10)	FICB and 3-in-1 block groups had lower VAS than the control group at 0 and 2 hours postoperatively (*P* < .05). There was no difference between any of the groups at the 4th, 6th, and 24th hour.
Temelkovska- Stevanovska et al^21^	2014	Prospective randomized	Extracapsular hip fracture	30 continuous 3-in-1 versus 30 FICB	Verbal descriptive scale (0-4)	No difference at 1, 2, and 12 hours. 3-in-1 significantly better at 24, 36, and 48 hours (*P* = .00001).
Newman et al^20^	2013	Prospective randomized	Femoral neck fracture	51 3-in-1 versus 56 FICB	VAS (100-mm scale)	3-in-1 block provided greater reduction in VAS than FICB (*P* = .047). Patients receiving femoral nerve block required less morphine than those receiving FICB (*P* = .041).

Abbreviations: FICB, fascia iliaca compartment block; GA, general anesthesia; VAS, Visual Analog Scores.

## Preoperative Pain Control With FICB

Thirteen papers were identified pertaining to the preoperative use of FICB, collectively indicating that FICB decreases pain scores, reduces opioid usage, and facilitates positioning for spinal anesthesia in hip fracture patients. In a study analyzing the efficacy of FICB, Lopez et al noted 1 block failure out of 27. Some of the patients did not receive any other pain medication prior to entering the operating room.^26^ Kumar et al described the successful use of the FICB in hip fractures to provide a safe method of controlling preoperative pain.^27^ Multiple studies have shown that FICB can be effectively administered in the ED setting, decreases preoperative pain scores, and reduces opioid requirements.^16,28-30^ Williams et al showed that preoperative administration of FICB decreases opioid use and effectively prevents opioid overdose in hip fracture patients.^31^ Høgh et al and Hanna et al specifically looked at FICB administered by junior residents in the ED and found significantly decreased pain scores and improved pain-free range of motion, suggesting that FICB can be effectively administered even by newly trained junior residents.^14,32^ A study on paramedic-administered FICB found that prehospital nerve block was realistically achievable and significantly reduced pain scores.^33^

Research has also been done to determine the utility of FICB in facilitating patient positioning for spinal anesthesia. Diakomi et al found that FICB not only reduced preoperative pain scores and patient satisfaction but also reduced time required for spinal block.^34^ In an Randomized Controlled Trial (RCT) comparing FICB with intravenous alfentail, Yun et al demonstrated superiority of FICB in pain reduction and positioning time for spinal anesthesia.^35^ Candal-Couto et al demonstrated significantly increased passive range of motion after administration of FICB.^36^ Thus, FICB seems to not only decrease pain but also facilitate administration of spinal anesthesia through increasing passive range of motion (Table 2).

**Table 2.** Summary of Studies on the Preoperative Use of FICB in Patients With Hip Fracture.

Study	Year	Method	Fracture	Comparison	Primary Outcome	Results
Kumar et al^2^ ^7^	2016	Prospective observational	Unspecified hip fracture	50 FICB pre-block versus post-block	VAS (0-10)	Post-block VAS (2.94) significantly less than pre-block (7.5), *P* < .01.
Williams et al^3^ ^1^	2016	Prospective cohort	Femoral neck fracture	50 FICB versus 69 standard analgesia	VAS (0-10)	VAS was significantly lower for FICB than standard analgesia alone (*P* = .001)
Groot et al^16^	2015	Prospective observational	Unspecified hip fracture	43 FICB pre-block versus post-block	10-point numerical rating scale (NRS)	Significant reduction in NRS following FICB (*P* = .04)
McRae et al^3^ ^3^	2015	Prospective randomized	Suspected femoral or hip fracture	11 FICB versus 13 standard care	11-point NRS	FICB had a greater reduction in NRS than standard care group (50% vs 22%, *P* = .025) after 15 minutes
Diakomi et al^3^ ^4^	2014	Prospective randomized	Unspecified hip fracture	21 FICB versus 20 IV fentanyl	Pain during positioning, NRS (0-10)	FICB showed significantly lower NRS scores (*P* < .001)
Hanna et al^14^	2014	Prospective cohort	Unspecified hip fracture	52 FICB versus 52 standard care	VAS (0-10)	VAS significantly lower (*P* ≤ .05) at 2 and 8 hours post-block
Haines et al^28^	2012	Prospective observational	Intertrochanteric and femoral neck fractures	20 FICB pre-block versus post-block	VAS (0-10)	Significantly less pain at all time points post-block (*P* < .029)
Elkhodair et al^2^ ^9^	2011	Prospective observational	Femoral neck fracture	137 FICB pre-block versus post-block	VAS (0-10)	Successful block (VAS reduction of ≥3 points) was achieved in 77.4% of cases in the ED
Yun et al^35^	2009	Prospective randomized	Femoral neck fracture	20 FICB versus 20 IV alfentanil	VAS (0-10), time to achieve spinal anesthesia	FICB group had lower pain scores during positioning (*P* = .001) and faster initiation of spinal anesthesia (6.9 ± 2.7 minutes vs 10.8 ± 5.6 minutes; *P* = .009)
Høgh et al^32^	2008	Prospective observational	Unspecified hip fracture	187 FICB pre-block versus post-block	Verbal pain score (0-4), passive range of motion	Median passive hip flexion increased from 15° to 36° at 1 hour after FICB (*P* = .03). Median pain scores were significantly reduced after FICB (*P* = .021)
Foss et al^37^	2007	Prospective randomized controlled	Unspecified hip fracture	24 FICB versus 24 intramuscular morphine	Verbal pain score (0-4), morphine requirement	FICB group had lower pain scores at rest (*P* < .01) and with movement (*P* = .02). Median total morphine consumption was 0 mg for FICB versus 6 mg for morphine group (*P* < .01)
Monzon et al^30^	2007	Prospective randomized controlled	Unspecified hip fracture	62 FICB versus 92 IV NSAID (diclofenac or ketorolac)	VAS (0-10)	Average VAS was lower in NSAID group at 15 minutes (*P* < .001). There was no significant difference at 2 hours (*P* = .764) and 8 hours (*P* = .083)
Candal-Couto et al^36^	2005	Prospective observational	Femoral neck fracture	30 FICB pre-block versus post-block	VAS (0-10), passive hip flexion	VAS decreased from 7.2 to 4.6 after FICB. There was a mean increase in passive hip flexion of 44°

Abbreviations: ED, emergency department; DSM-IV, Diagnostic and Statistical Manual of Mental Disorders, Fourth Edition; FICB, fascia iliaca compartment block; IV, intravenous; NSAID, nonsteroidal anti-inflammatory medication; VAS, visual analog scores.

## Nonsteroidal Anti-Inflammatory Medications Versus FICB in Geriatric Patients

In order to reduce opioid use in the geriatric population, NSAIDs are often used in hip fracture patients. In a prospective trial comparing NSAIDs with FICB, Fujihara et al found patients treated with FICB to have significantly decreased VAS for up to 8 hours after administration compared to those treated with parenteral NSAIDs.^38^ In another study by Monzón et al, patients receiving parenteral NSAIDs had decreased pain scores compared to those receiving FICB at 15 minutes, but there was no difference between the 2 groups at 2- and 8-hour assessment.^39^

## Postoperative Pain Control With FICB

Six papers were identified demonstrating that FICB effectively decreases pain scores, opioid use, and opioid-related side effects in patients with hip fracture. Nie et al showed that patients receiving continuous FICB after hip arthroplasty had significantly decreased pain scores and fewer side effects than those receiving patient-controlled analgesia with opioids.^17^ Bang et al also performed a prospective, randomized trial of patients who received FICB versus patient-controlled analgesia in postoperative hemiarthroplasty patients and found visual analog scales were similar in both groups; however, opioid use was significantly lower in the block group.^40^ A 2002 Cochrane review demonstrated appropriately based nerve block reduced the amount of oral and parenteral analgesics postoperatively.^41^ Foss et al performed a double-blinded study where 1.0% mepivacaine was injected into the fascia iliaca block versus normal saline and found a reduction in opioid consumption in the fascia iliac block cohort.^37^ Monzon et al noted a drastic reduction in pain after FICB was administered and more importantly a beneficial effect of preventing delirium.^29^ A study by Mouzopoulos et al stratified patients with hip fracture into 3 groups based on risk of postoperative delirium and then compared delirium occurrence in patients receiving FICB to those receiving standard analgesia. The study found that FICB decreased the risk of postoperative delirium in intermediate but not high- or low-risk patients.^42^ Dulaney-Cripe et al and Mangram et al demonstrated that continuous FICB through an indwelling catheter produced decreased opioid usage and shorter length of stay in the hospital.^43,44^ Taken together, these results show that FICB reduces postoperative pain in hip fracture patients, thereby decreasing opioid consumption (Table 3). Decreased opioid consumption may reduce the incidence of delirium in susceptible patients leading to shorter mean hospital stays with fewer complications.

**Table 3.** Summary of Studies on the Postoperative Use of FICB in Patients With Hip Fracture.

Study	Year	Method	Fracture	Comparison	Primary Outcome	Results
Bang et al^40^	2016	Prospective randomized controlled	Femoral neck fracture	11 FICB + patient-controlled analgesia (PCA) versus 11 PCA alone	VAS (0-10)	There was no significant difference in VAS. Mean fentanyl requirement was 246.3 μg in the FICB group versus 351.4 μg in the control group
Nie et al^1^ ^7^	2015	Prospective randomized	Femoral neck fracture	53 FICB versus 53 PCA	Numerical rating score (0-10), postoperative delirium	FICB group had lower pain scores (*P* = .039). 10 patients in PCA group developed delirium versus 5 in FICB group
Mangram et al^44^	2015	Retrospective case–control	Unspecified hip fracture	44 FICB versus 64 standard analgesia care	VAS (0-10)	FICB group had lower pain scores at 4, 8, and 20 hours (*P* < 0.05). 68.2% of FICB group had improved pain control versus 31.8% of standard care group at 12.5 hours
Fujihara et al^3^ ^8^	2013	Prospective randomized	Femoral neck fracture	31 FICB versus 25 diclofenac suppository	VAS (0-100)	FICB group had lower VAS upon arrival in recovery, at 6 hours, and at 12 hours after surgery (*P* < .05)
Mouzopoulos et al^42^	2009	Prospective randomized controlled	Femoral neck and intertrochanteric fracture	102 FICB versus 105 placebo	Delirium (assessed by *DSM-IV* and Confusion Assessment Method Criteria)	The relative risk of delirium was 0.45 (CI: 0.23-0.87) in the FICB group compared to placebo

Abbreviations: CI, confidence interval; FICB, fascia iliaca compartment block; VAS, Visual Analog Scores.

Other research has looked at the use of nerve blocks as an adjunct mode of analgesia in children and adolescents with hip fractures. A prospective cohort study by Black et al showed that FICB provides effective analgesia, reduces systemic opioid requirements, and has fewer side effects than morphine in children and adolescents with hip fractures.^45^ These data suggest that nerve blocks may play an important role in analgesic management of the pediatric population as well.

## Neuroanatomy of Localized Nerve Blocks for Hip Fracture

The importance of an accurate anatomical understanding of the sensory innervation of the hip can be demonstrated by the growing body of literature, suggesting alternatives to pain control with opioid analgesics. The sensory innervation of the hip capsule has been well defined in the literature. In one of the original cadaveric studies detailing hip neuroanatomy, Birnbaum et al noted that the hip capsule is innervated not only by the obturator nerve but also by branches of the femoral, superior gluteal, and the sciatic nerves. The authors suggested that a nerve block which targeted multiple of these sensory nerves would be the most effective in providing local anesthesia to the hip.^46^ In a recent review of 17 different neuroanatomical studies of the hip, Simons et al found that the hip is supplied by the femoral, obturator, sciatic, and superior gluteal nerves, as well as the nerve to the quadratus femoris. The anterior aspect of the hip capsule is the most densely innervated, suggesting that periarticular infiltration of this area during arthroplasty can play a significant role in reducing postoperative pain. The authors also noted that infiltration of the anterior aspect of the labrum from 10- to-2 o’clock position may be useful as this is a highly innervated area as well.^47^ Although the innervation of the hip capsule seems fairly well defined in the literature, there are limited data on the innervation of the trochanteric area of the femur. Only 1 rigorous anatomical study was identified during literature review.

**Figure 1.**
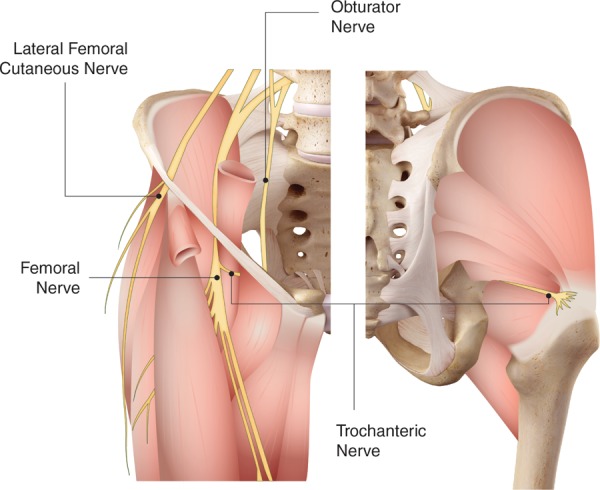
Anatomical diagram of the innervation of the hip.

Genth et al performed 7 dissections on adult cadavers to evaluate the innervation of the trochanteric region of the femur. The authors were unable to find any histologic evidence of nerve contributions to the trochanteric region from the inferior gluteal nerve, the superior gluteal nerves, and the sciatic nerve. They did note a nerve branch running with the medial femoral circumflex artery which passed medial to iliopsoas and lateral to pectineus as a neurovascular bundle toward the greater trochanteric region (Figure 1). They concluded that innervation to the greater trochanter is likely supplied entirely by this single nerve branch, which originates from the femoral nerve. Their histologic analysis demonstrated mostly unmyelinated fibers, with a smaller composition of myelinated fibers consistent with what would be found in a sensory nerve.^19^ Taken together, these studies indicate that localized sensory block of the femoral nerve may not only decrease pain from the hip capsule itself but may also provide anesthesia to the trochanteric area of the hip. More research is necessary to better delineate the neuroanatomy of this area.

Although the efficacy of FICB has been well established in the literature, there is disagreement about the exact neuroanatomy targeted by the block. In a comparison of 3-in-1 femoral nerve block and FICB, Capdevila et al found that the obturator nerve was effectively blocked in 38% of patients receiving FICB. Radiographic analysis showed that anesthesia was achieved through concurrent internal and external spread of the local anesthetic solution under the fascia iliaca and between the iliacus and psoas muscles in 62 of 92 blocks. Isolated spread of anesthesia under the fascia iliaca was achieved in only 36% of patients undergoing FICB.^25^ In contrast to this, a more recent study on the distribution of anesthetic following FICB using MRI by Swenson et al found no evidence of anesthetic spread to the obturator nerve. Additionally, there was no adductor weakness in any of the 5 patients studied. Magnetic resonance imaging analysis of FICB showed isolated spread of anesthetic over the surface of the iliacus and psoas without infiltration of the muscle layers.^48^ The authors also performed several cadaveric dissections to delineate the fascia iliaca compartment. Because the iliac fascia is tightly adherent to underlying structures, the site of anesthetic injection in FICB is a potential space and there is a wide margin for error. Swenson et al suggested that variability in anesthetic distribution seen in prior studies could be explained by inconsistent penetration of the iliac fascia, as these studies did not utilize ultrasound guidance.^49^

Another study by Dolan et al specifically examined ultrasound guidance in FICB. The authors found that ultrasound-guided FICB was more effective than the loss of resistance technique in achieving femoral and obturator nerve blocks, as defined by anesthesia of the medial thigh. Ultrasound guidance improved sensory block to the medial thigh from 60% to 95%. In contrast to Swenson et al, however, the authors argued that more consistent injection of anesthetic in the fascia iliaca compartment using ultrasound guidance allowed for spread of anesthetic medially to the obturator nerve.^49^ Finally, a study by Morau et al suggested that although FICB provided effective sensory anesthesia of the medial thigh, this was not equivalent to obturator nerve block. These authors noted that electromyography or hip adductor strength might be more accurate than sensory anesthesia in assessing obturator block in patients undergoing FICB.^50^ These studies show that there is still significant debate surrounding the neuroanatomic basis of FICB-administered anesthesia and illustrate the need for further research in this area.

## Conclusion

Based on our review of the current literature, the need for better pain management in geriatric patients is readily apparent. Localized nerve blocks present exciting possibilities for achieving anesthesia in geriatric patients while minimizing adverse side effects such as delirium and constipation. Although there seems to be no clear consensus in the literature about whether FICB or 3-in-1 femoral block is more effective, FICB is an appealing option due to its relative ease of administration and reproducibility. The neuroanatomic basis for FICB seems to be the local spread of anesthetic across the fascia iliaca to anesthetize branches of the femoral nerve that extend to the trochanteric area and to the hip capsule. There is conflicting evidence on the efficacy of FICB in anesthetizing the obturator nerve. Additional research examining the neuroanatomy of nerve blocks in the hip is necessary to provide more effective and reliable anesthesia. With future research, FICB and other nerve blocks will continue to become an invaluable adjunct to systemic analgesia in geriatric patients with hip fractures, leading to decreased opioid side effects and improved long-term outcomes.
